# Overexpression of Wnt5a Promotes Angiogenesis in NSCLC

**DOI:** 10.1155/2014/832562

**Published:** 2014-06-05

**Authors:** Lingli Yao, Baocun Sun, Xiulan Zhao, Xueming Zhao, Qiang Gu, Xueyi Dong, Yanjun Zheng, Junying Sun, Runfen Cheng, Hong Qi, Jindan An

**Affiliations:** ^1^Department of Pathology, Tianjin Medical University, Tianjin 300070, China; ^2^Department of Pathology, Affiliated Anhui Provincial Hospital, Anhui Medical University, Hefei, Anhui 230001, China; ^3^Department of Pathology, Tianjin Cancer Hospital, Tianjin Medical University, Tianjin 300060, China; ^4^Department of Pathology, Tianjin General Hospital, Tianjin Medical University, Tianjin 300052, China

## Abstract

To evaluate Wnt5a expression and its role in angiogenesis of non-small-cell lung cancer (NSCLC), immunohistochemistry and CD31/PAS double staining were performed to examine the Wnt5a expression and we analyze the relationships between Wnt5a and microvessel density (MVD), vasculogenic mimicry (VM), and some related proteins. About 61.95% of cases of 205 NSCLC specimens exhibited high expression of Wnt5a. Wnt5a expression level was upregulated in the majority of NSCLC tissues, especially in squamous cell carcinoma, while its expression level in adenocarcinoma was the lowest. Wnt5a was also found more frequently expressed in male patients than in female patients. Except for histological classification and gender, little association was found between Wnt5a and clinicopathological features. Moreover, Wnt5a was significantly correlated with prognosis. Overall, Wnt5a-positive expression in patients with NSCLC indicated shorter survival time. As for vascularization in NSCLC, Wnt5a showed close association with VM and MVD. In addition, Wnt5a was positively related with **β**-catenin-nu, VE-cadherin, MMP2, and MMP9. The results demonstrated that overexpression of Wnt5a may play an important role in NSCLC angiogenesis and it may function via canonical Wnt signal pathway. This study will provide evidence for further research on NSCLC and also will provide new possible target for NSCLC diagnosis and therapeutic strategies.

## 1. Introduction

In recent years, primary lung cancer occupies the leading cause of cancer mortality in the world [1]. Among primary lung cancer cases, approximately 80% of the cases are non-small-cell lung cancer [1, 2]. Although recent advancements have improved the survival rate of NSCLC patients [3–5], the high mortality related to NSCLC remains a daunting challenge [6]. Therefore, it is important to pay more attention to clarifying the mechanism of tumor biology in order to improve the prognosis of NSCLC patients. Indeed, angiogenesis theory has contributed significantly to tumor research.

Angiogenesis theory believes that tumor angiogenesis is essential for tumor growth and metastasis. When solid tumor grows to more than 2 mm in diameter, it needs to induce the generation new blood vessels to obtain a continuous supply of oxygen and nutrition to maintain its growth; otherwise, it would result in necrosis due to ischemia and anoxia [7, 8]. Thus, antiangiogenesis has become hot topic on tumor research. In fact, Wnt signaling pathway has been proven to be involved in this theory [9, 10].

Wnt5a is an important regulator of Wnt signaling pathway and has been demonstrated to play an important role in lung development and tumorigenesis [11, 12]. However, the biologics of Wnt5a in human cancers are still unclear. On the one hand, Wnt5a was found frequently upregulated in various cancers, including breast cancer, pancreatic cancer, prostate cancer, and gastric cancer [13–16]. On the other hand, Wnt5a was reported as a tumor suppressor gene in several cancers [12, 17, 18]. In addition, Wnt5a was proven to contribute to vascularization of embryonic stem cells [19]. Although Wnt5a was considered as an oncogene in lung cancer [20], its role in angiogenesis of lung cancer is still ambiguous.

Herein, the present study would investigate the expression of Wnt5a and its role in angiogenesis of human NSCLC. First, immunohistochemistry was performed to examine the Wnt5a expression in 205 NSCLC tissues. Then, the relationship between Wnt5a and microvessel density (MVD) was detected. In addition, the relationship between Wnt5a and vasculogenic mimicry (VM), a special blood supply mode, was also detected. Finally, correlations between Wnt5a and expressions of some angiogenesis-related proteins and *β*-catenin were analyzed.

## 2. Materials and Methods

### 2.1. Patients

Tissue specimens were obtained from 205 patients who had undergone surgical resection for lung cancer in Tianjin Medical University Cancer Institute and Hospital from October 1990 to November 2010. The 205 NSCLC samples were composed of 79 cases of squamous cell carcinoma, 75 cases of adenocarcinoma, and 51 cases of large cell lung cancer. The diagnoses of these samples were independently verified by two pathologists according to the standards of classification [2, 21]. The average age of the patients at the time of diagnosis was 59.1 years (30 years to 88 years). The data of clinicopathological parameters were harvested from the patients' clinical records and pathological reports. Time to death, final follow-up examination, and diagnosis of metastasis were recorded from the date of surgery. This study was approved by the Ethical Committee of Tianjin Medical University prior to its initiation.

### 2.2. Immunohistochemistry and CD31/Periodic Acid Schiff (PAS) Double Staining

This assay was performed as described by Zhang et al. [22] and Sun et al. [23, 24]. The tissues were 10% formalin-fixed, paraffin-embedded, and cut into 4 *μ*m thickness. All slides were then deparaffinized in xylene and dehydrated with descending-grade alcohol. Endogenous peroxidase activity was quenched by brooding in methanol containing 3% hydrogen peroxide for 30 min at room temperature. After blocking with recommended serum for 20 min at room temperature, the slides were incubated with a primary antibody overnight at 4°C and a homologous secondary antibody for 1 h at room temperature in a humidified box. Then the sections were stained with freshly dispensed diaminobenzidine solution (DAB) for observation under a microscope. In the process, the slides were all rinsed three times in phosphate-buffered saline (PBS) (pH 7.2) before each step, except for the procedure of serum blocking to incubation with the primary antibody. The slides were then counterstained with hematoxylin, dehydrated with ascending-grade ethanol, air-dried, cleared with xylene, and mounted. For CD31/periodic acid Schiff (PAS) double staining, the sections were still incubated with 1% periodic acid for 15 min and Schiff reagent for observation under a microscope at 37°C between DAB staining and hematoxylin counterstaining. In this process, distilled water instead of PBS was used for washing.

In the current study, the primary antibodies to Wnt5a and VE-cadherin were purchased from Abcam (Cambridge, UK). Antibodies to CD31, CD34, *β*-catenin, and MMP2 were from Invitrogen Zymed Laboratories (San Diego, USA). Antibody to MMP9 was from Santa Cruz Biotechnology (Santa Cruz, CA, USA). Positive control and negative control were performed for each batch. For the negative control, PBS was used instead of the primary antibody. For the positive control, a foregone positively expressed tissue section was used.

The results were evaluated following the method described by Bittner et al. [25]. The percentage and the intensity of the positive cells were both measured. The percentage was stratified as follows: 0 for less than 5% positive cells, 1 for less than 30% positive cells, 2 for less than 60% positive cells, and 3 for more than 60% positive cells. The intensity was also classified as follows: 0 (negative), 1 (weak), 2 (moderate), and 3 (strong). The sum of positive cell and staining intensity scores, which was more than 3 for the final result, was considered as the positive sample for each slide. Positive sample of *β*-catenin nuclear expression was deemed as those tissues which lost consecutive membrane location and acquired nuclear location more than 10% cells. MVD was determined from CD34-stained sections at the hot spot through light microscopy examination. The fields with the greatest neovascularization were examined by scanning tumor sections at low power (×100). The average vessel count of the five fields (×200) was regarded as the MVD.

### 2.3. Statistical Analysis

All data in the study were evaluated with SPSS17.0 software (SPSS, Chicago, IL, USA). Survival data were analyzed according to Kaplan-Meier test. Differences in survival curves were assessed by the log rank test. Crosstabs, Pearson *χ* ^2^ test, and Spearman correlation analysis were used as needed. All *P* values were two-sided, and *P* < 0.05 was considered statistically significant.

## 3. Results

### 3.1. Association of Wnt5a with Clinicopathological Features in Human NSCLC

**Figure 1:**
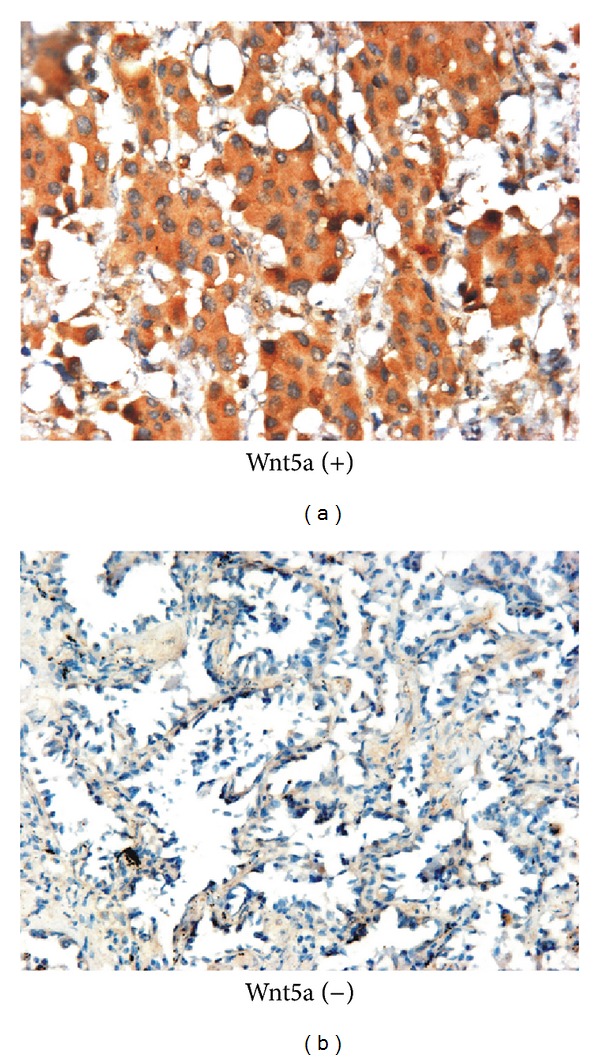
The expression of Wnt5a in NSCLC tissues (immunohistochemical staining, ×200). Left photo showed that Wnt5a was positively expressed in the cytoplasm of tumor cells while Wnt5a was negatively expressed in the tumor cells at the right picture.

Wnt5a positive expression appeared as brown granules staining in the cytoplasms of the tumor cells. Among 205 NSCLC specimens, Wnt5a was detected in 127 cases (Figure 1). Approximately 61.95% of NSCLC exhibited high expression of Wnt5a. According to Wnt5a presence, all samples were divided into two groups: Wnt5a-positive group (*n* = 127) and Wnt5a-negative group (*n* = 78). Then, the relationship between Wnt5a and clinicopathological features was analyzed separately. Statistical data in Table 1 showed that Wnt5a was significantly associated with histological classification and gender (*P* = 0.016 and 0.012, resp.). Among the three histological types, Wnt5a was frequently expressed in squamous cell carcinoma (70.89%, 56/79), while Wnt5a-positive expression in adenocarcinoma was the lowest (49.33%, 37/75). In male samples, Wnt5a was found expressed more than in female patients (67.59%, 98/145* versus* 48.33%, 29/60). However, little correlation was found between Wnt5a-positive expression and other clinicopathological characteristics, such as age, tumor size, location, histological differentiation, pleura invasion, stage, metastasis, lymph node status, and therapy before surgery (*P* > 0.05, Table 1).

**Figure 2:**
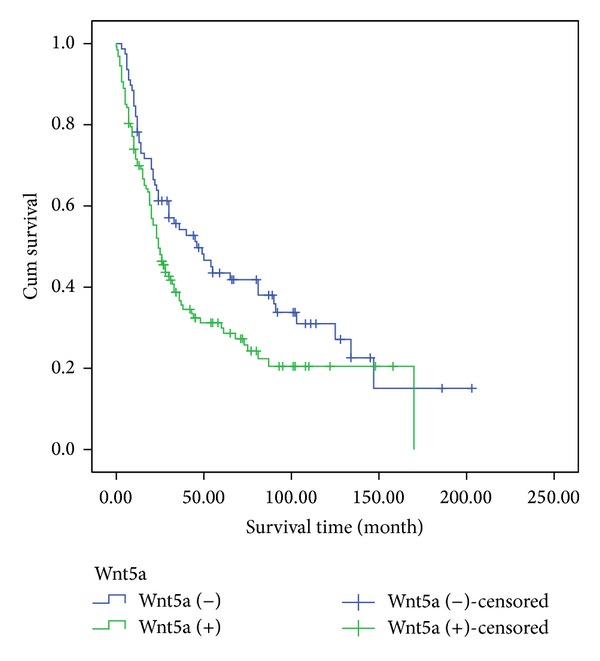
Result of the Kaplan-Meier survival analysis. Kaplan-Meier survival analysis showed that Wnt5a-positive patients have shorter survival periods than Wnt5a-negative patients (*P* = 0.026).

To verify the clinical significance of Wnt5a, all 205 NSCLC patients were followed up and the relationship between their outcomes and Wnt5a expression was examined. Statistical analysis showed that the overall survival period of Wnt5a-positive patients was enormously shorter than that of Wnt5a-negative patients (*P* = 0.026). The mean survival period of Wnt5a-negative group was 52.31 months, whereas that of Wnt5a-positive group was only 33.64 months (Figure 2).

### 3.2. Association of Wnt5a with Angiogenesis in Human NSCLC

**Figure 3:**
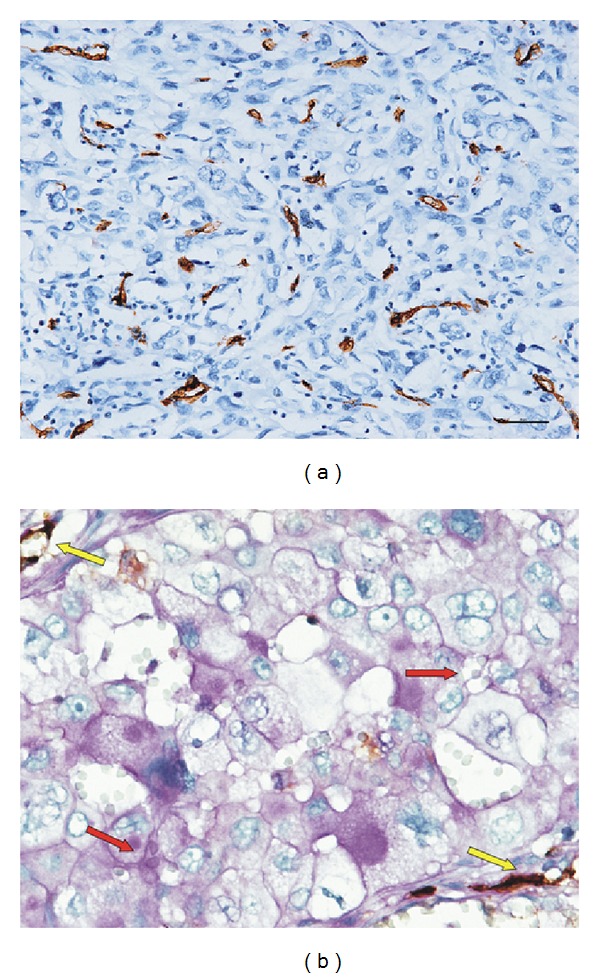
The angiogenesis status in NSCLC. (a) MVD staining for CD34 in NSCLC (immunohistochemical staining, ×200). A hotspot with high MVD was positively stained. (b) CD31/PAS double staining for VM (×400). The VM channel showed a positive expression for PAS but a negative expression for CD31 (red arrow). The endothelial channel showed positive expressions for both CD31 and PAS (yellow arrow).

To evaluate the role of Wnt5a in angiogenesis of NSCLC, relationships between MVD, VM, and Wnt5a were examined. CD34 was stained to calculate the MVD and CD31/PAS double staining was recruited to identify the VM (Figure 3). According to the median value of MVD or the presence of VM, all 205 NSCLC cases were classified as high CD34-MVD (≥28, *n* = 113) or low CD34-MVD (<28, *n* = 92) and divided into VM group (*n* = 28) or non-VM group (*n* = 177). As shown in Table 2, significant correlation was found between Wnt5a and VM (*P* = 0.021, *r* = 0.165), as well as Wnt5a and CD34-MVD (*P* = 0.026, *r* = 0.157).

### 3.3. Association of Wnt5a with Angiogenesis Related Proteins in Human NSCLC

**Figure 4:**
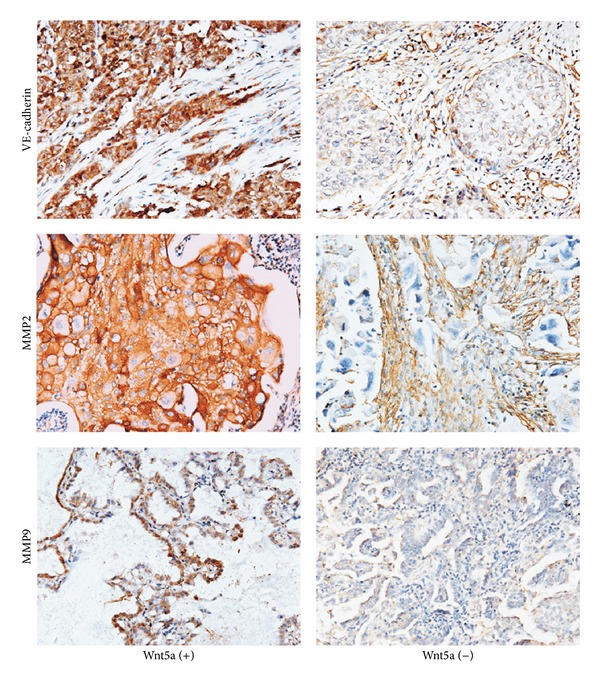
The expressions of some related proteins in NSCLC (immunohistochemical staining, ×200). In Wnt5a-positive group, positive expressions of VE-cadherin, MMP2, and MMP9 were all located in the cytoplasm of tumor cells. However, these proteins were negatively expressed in NSCLC tissues of Wnt5a-negative group. VE-cadherin positive expression in endothelial cells or MMPs positive expression in stromal cells could provide an internal positive control for both, respectively.

To further investigate the angiogenesis of NSCLC, some related proteins (VE-cadherin, MMP2, and MMP9) were also examined in this study. Positive expressions of these proteins were all located in the cytoplasm of tumor cells (Figure 4). Among 205 NSCLC tissues, VE-cadherin was positively expressed in 101 specimens (49.27%, 101/205), while MMP2 and MMP9 were found positively expressed in 67 cases (32.68%, 67/205) and 80 samples (39.02%, 80/205), respectively. Moreover, the relationships between Wnt5a and these related proteins were also studied. VE-cadherin was found closely related with Wnt5a (*P* = 0.004, *r* = 0.210) (Table 2). VE-cadherin-positive and Wnt5a-positive samples included 73 cases, and both negative samples included 50 cases. Similar to VE-cadherin, MMP2 and MMP9 both showed the remarkable relevance with Wnt5a (*P* < 0.001, *r* = 0.268; *P* = 0.003, *r* = 0.215, resp.) (Table 2).

### 3.4. Association of Wnt5a with *β*-Catenin Nuclear Expression in Human NSCLC

**Figure 5:**
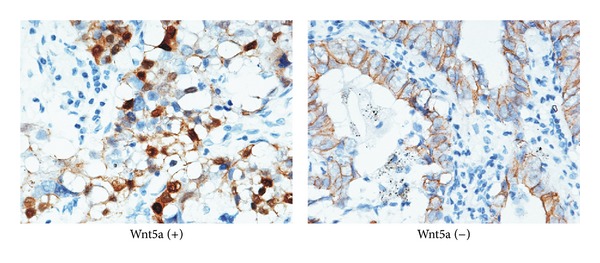
The expression of *β*-catenin in NSCLC (immunohistochemical staining, ×400). In Wnt5a-negative tissues, *β*-catenin exhibited the continuous expression in membrane, while in Wnt5a-positive group, *β*-catenin lost its continuous membrane expression and translocated to nucleus.

To elucidate the possible mechanism of Wnt5a in angiogenesis of human NSCLC, *β*-catenin nuclear expression was detected and its relationship with Wnt5a was analyzed (Figure 5). In the current study, positive *β*-catenin nuclear expression appeared in 32 cases of 205 NSCLC tissues (15.61%, 32/205). Wnt5a was shown to be closely associated with *β*-catenin nuclear location (*P* = 0.017, *r* = 0.171). Both Wnt5a and *β*-catenin nuclear locations were detected positively in 26 cases, while Wnt5a and *β*-catenin nuclear locations were both negatively expressed in 72 samples. Wnt5a-positive expression but *β*-catenin-nu negative location was found in 101 tissues, whereas *β*-catenin-nu positive and Wnt5a-negative samples included 6 cases (Table 2).

## 4. Discussion

The Wnt proteins family includes at least 19 secreted cysteine-rich glycoproteins that are involved in the regulation of a wide variety of normal and pathologic processes, including embryogenesis, differentiation, and tumorigenesis [26–30]. As one of the important members in the large Wnt family, Wnt5a has been shown to have close correlation with various cancers [13–15, 18].

In this study, expression of Wnt5a was investigated by immunohistochemistry in a large cohort of 205 human NSCLC tissues. The results showed that Wnt5a was upexpressed in the majority of cases. Except for histological classification and gender, Wnt5a-positive expression was found to exhibit little correlation with clinicopathological parameters. Wnt5a was more frequently expressed in squamous cell carcinoma. Our result was in accordance with the previous report. Huang and his colleagues examined Wnt5a expression in 123 NSCLC cases and found a similar result [20]. We also found that Wnt5a was more often expressed in male NSCLC patients, although none of the previous studies examined the relation between Wnt5a and gender in NSCLC [20, 31]. However, two studies strongly supported our result. Heikkila et al. reported that deficiency of Wnt5a could result in sex reversal, infertility, and/or malformation of the internal and external genitals [32]. In addition, Kovalchuk et al. concluded that Wnt signaling pathway, including Wnt5a, was differently induced between male and female mice after chronic radiation exposure [33]. Thus, we supposed that it may be related with sex hormone. Moreover, our data showed that overall survival time of those NSCLC patients with Wnt5a-positive expression was shorter than that of Wnt5a negative-expressed patients, which supported the results of Huang et al. and Nakashima et al. [20, 31]. Therefore, it may provide a good marker for clinical diagnosis and prognosis of NSCLC.

It is well known that a continuous supply of oxygen and nutrition is crucial for indefinite growth of solid tumors. Angiogenesis plays a vital role in development of tumor. Huang et al. investigated the relationship between Wnt5a and angiogenesis in 123 NSCLCs which included 67 cases of adenocarcinoma (AC), 50 cases of squamous cell carcinoma (SCC), and 6 cases of large cell carcinoma (LCC). They reported that the intratumoral Wnt5a expression was significantly correlated with the stromal expression of VEGF-A but insignificantly correlated with intratumoral microvessel density [20]. In this study, we also examined the relationship between Wnt5a and angiogenesis in NSCLC. Firstly, we evaluated the relation between Wnt5a and MVD, which is a classic marker of tumor angiogenesis. However, contrary to previous results [20], we found that there was significant correlation between Wnt5a and MVD in the present study. We supposed that it is due to the difference of object. Our study contained more samples including 205 NSCLC specimens. Moreover, the ratio for LCC was larger than Huang's study. As we know, LCC is a more progressive tumor in NSCLC. In addition, they only reported the relationship between Wnt5a and MVD, which only reflected the endothelial dependent vessel in the tumor. However, besides endothelial dependent vessel, Maniotis detected a novel blood supply mode, named vasculogenic mimicry (VM), in highly aggressive uveal melanomas [34]. Subsequently, many researchers have found VM in several malignant tumors [22, 35–40]. Thus, we also analyzed the correlation between Wnt5a and VM, which could reflect the blood supply of tumor at some degree. Statistical analysis showed that Wnt5a was positively correlated with VM. Wnt5a may play an important role in angiogenesis of NSCLC.

To further investigate the relationship between Wnt5a and angiogenesis, we conducted immunohistochemistry to examine expressions of some related proteins and analyzed their associations with Wnt5a. VE-cadherin, MMP2, and MMP9 have been proven to participate and represent partially VM [35, 39, 41]. Our data showed that Wnt5a was also positively associated with all the above proteins. The results further demonstrated that Wnt5a play an active role in angiogenesis of NSCLC.

We also detected *β*-catenin nuclear expression in NSCLC tissues and found it was positively correlated with Wnt5a. *β*-Catenin is a vital molecule of canonical Wnt signal pathway. Normally, *β*-catenin shows continuous membrane location, and while canonical Wnt pathway is activated, *β*-catenin shows translocation to nucleus. Thus, *β*-catenin nuclear expression is considered as an important marker for activated canonical Wnt pathway. Therefore, our data indicated that Wnt5a may function through canonical Wnt pathway. Though it was a member of noncanonical Wnt pathway, Wnt5a was reported to play a role in the canonical Wnt/*β*-catenin signaling pathway. The Wnt5a protein can act via Frz-5 receptor to initiate an intracellular pathway leading to the accumulation of *β*-catenin [20, 42].

## 5. Conclusions

Taken together, the current study demonstrated that Wnt5a was overexpressed in human NSCLC tissues and closely associated with tumor angiogenesis. Enhanced expression of Wnt5a may induce the nuclear accumulation of *β*-catenin and activate the canonical Wnt signaling pathway, thus leading to the upexpression of VE-cadherin, MMP2, and MMP9, then resulting in angiogenesis, and ultimately promoting the growth and metastasis of NSCLC. Therefore, this study may contribute to the mechanism of NSCLC research and provide new hope for NSCLC diagnosis and therapeutic strategies.

## Figures and Tables

**Table 1:** Correlation between Wnt5a and clinicopathological features in NSCLC.

Variant	Total	Wnt5a		*x* ^2^	*P* value
	*n*	Negative (%)	Positive (%)		
Gender					
Male	145	47 (32.41)	98 (67.59)	6.674	0.012
Female	60	31 (51.67)	29 (48.33)		
Age (yr)					
<60	101	37 (36.63)	64 (63.37)	0.169	0.774
≥60	104	41 (39.42)	63 (60.58)		
Size (cm)					
<3	29	11 (37.93)	18 (62.07)	0.000	1.000
≥3	176	67 (38.07)	109 (61.93)		
Location					
Center	106	38 (35.85)	68 (64.15)	0.451	0.565
Peripheral	99	40 (40.40)	59 (59.60)		
Histological classification					
SCC	79	23 (29.11)	56 (70.89)	8.222	0.016
AC	75	38 (50.67)	37 (49.33)		
LCC	51	17 (33.33)	34 (66.67)		
Differentiation					
Well	35	17 (48.57)	18 (51.43)	2.044	0.360
Moderate	87	32 (36.78)	55 (63.22)		
Poor	83	29 (34.94)	54 (65.06)		
Pleura invasion					
No	113	39 (34.51)	74 (65.49)	1.335	0.252
Yes	92	39 (42.39)	53 (57.61)		
Lymph node metastasis					
No	117	45 (38.46)	72 (61.54)	0.020	1.000
Yes	88	33 (37.50)	55 (62.50)		
T stage					
T1 + T2	149	56 (37.58)	93 (62.42)	0.050	0.872
T3 + T4	56	22 (39.29)	34 (60.71)		
Clinical stage					
I + II	158	62 (39.24)	96 (60.76)	0.415	0.609
III + IV	47	16 (34.04)	31 (65.96)		
Distant metastasis					
No	147	57 (38.78)	90 (61.22)	0.116	0.752
Yes	58	21 (36.21)	37 (63.79)		
Therapy before surgery					
No	186	69 (37.10)	117 (62.90)	0.772	0.458
Yes	19	9 (47.37)	10 (52.63)		

*P* < 0.05 means statistical significance.

**Table 2:** Relationship between Wnt5a and angiogenesis, expression of related proteins, and *β*-catenin nuclear expression in NSCLC.

Variant	Total	Wnt5a		*P* value	*r*
	*n*	Negative (%)	Positive (%)		
CD34-MVD					
<28	92	52 (56.52)	40 (43.48)	0.026	0.157
≥28	113	46 (40.71)	67 (59.29)		
VM					
Negative	177	73 (41.24)	104 (58.76)	0.021	0.165
Positive	28	5 (17.86)	23 (82.14)		
VE-cadherin					
Negative	104	50 (48.08)	54 (51.92)	0.004	0.210
Positive	101	28 (27.72)	73 (72.28)		
MMP2					
Negative	138	65 (47.10)	73 (52.90)	<0.001	0.268
Positive	67	13 (19.40)	54 (80.60)		
MMP9					
Negative	125	58 (46.40)	67 (53.60)	0.003	0.215
Positive	80	20 (25.00)	60 (75.00)		
*β*-Catenin nuclear expression					
Negative	173	72 (41.62)	101 (58.38)	0.017	0.171
Positive	32	6 (18.75)	26 (81.25)		

*P* < 0.05 means statistical significance.
